# Evaluating the Antimicrobial and Antibiofilm Efficacy of Lavender Essential Oil and Linalool on Dual *Candida albicans* Biofilms With *Staphylococcus aureus* and *Staphylococcus epidermidis* From Canine External Otitis

**DOI:** 10.1002/vms3.70407

**Published:** 2025-05-23

**Authors:** Navid Neisari, Aghil Sharifzadeh, Bahar Nayeri Fasaei, Sepideh Asadi, Alireza khosravi, Abolfazl Rafati Zomorodi, Javad Malakootikhah

**Affiliations:** ^1^ Department of Microbiology and Immunology Faculty of Veterinary Medicine University of Tehran Tehran Iran; ^2^ Department of Pathobiology, Faculty of Veterinary Medicine Amol University of Special Modern Technologies Amol Iran; ^3^ Department of Bacteriology and Virology School of Medicine Shiraz University of Medical Sciences Shiraz Iran; ^4^ Student Committee of Medical Education Development, Education Development Center Shiraz University of Medical Sciences Shiraz Iran

**Keywords:** antibiofilm, antimicrobial, essential oil, lavender, linalool

## Abstract

**Background:**

Biofilm formation significantly contributes to the rise of antimicrobial resistance, treatment failures and recurrent infections. Essential oils (EOs), particularly lavender EO (LEO), have gained attention for their antimicrobial and antibiofilm activities. This study investigates the effects of LEO and linalool on *Staphylococcus aureus* (*S. aureus*), *Staphylococcus epidermidis* (*S. epidermidis*), and *Candida albicans* (*C. albicans*) isolates.

**Materials and Methods:**

The chemical composition of LEO was analysed using gas chromatography‐mass spectrometry (GC‐MS). Eight clinical and reference microorganisms were tested, including four *C. albicans*, three *S. aureus*, and three *S. epidermidis* isolates, to assess their biofilm‐producing potential with the tissue microtiter plate method. Antimicrobial and antibiofilm activities of LEO and linalool were evaluated in planktonic, single‐biofilm, and dual‐biofilm phases through microbroth dilution and scanning electron microscopy (SEM).

**Results:**

The minimum inhibitory concentration (MIC) and minimum bactericidal/fungicidal concentration (MBC/MFC) of LEO in the planktonic phase were 1250 µg/mL and 2500 µg/mL against *Staphylococcus* isolates, respectively, while the corresponding value for *C. albicans* isolates was 5000 µg/mL. 90% biofilm inhibition was achieved at concentrations of 5000 µg/mL and 40,000 µg/mL for *Staphylococcus* and *C. albicans*, respectively. LEO completely inhibited dual biofilms formed by *C. albicans*/*S. aureus* and *C. albicans*/*S. epidermidis* at 20,000 µg/mL, whereas linalool attained 100% inhibition at 40,000 µg/mL.

**Conclusion:**

LEO demonstrates significant antimicrobial and antibiofilm activity against *Staphylococcus* and *C. albicans* isolates, effective in both planktonic and biofilm phases.

## Graphical abstract

The minimum inhibitory concentration of antimicrobial and anti‐biofilm activity of lavender essential oil (LEO) was determined. LEO exhibited robust antimicrobial and anti‐biofilm activity against clinically relevant *Staphylococcus* spp. and *Candida albicans* (*C. albicans*), including both planktonic and biofilm forms.

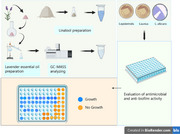

## 1 Introduction

Otitis externa (OE) is considered one of the most prevalent infections affecting dogs globally, with an estimated frequency ranging from 5% to 20% (Ponn et al. 2024). The clinical signs in OE cases include head shaking, purulent discharge, malodour, pain and swelling resulting from inflammation, as well as erythema of the pinnae, external meatus and lining of the external canal. In recurrent or chronic OE cases, clinical signs may progress to stenosis of the external ear canal and occlusion (Vercelli et al. 2021). Although bacteria and yeasts are not the primary causes of OE, they are responsible for persistent inflammation that contributes to the chronicity of the pathology (Secker et al. 2023).

*Staphylococcus spp*., *Pseudomonas spp*., *Corynebacterium spp*., *Proteus spp*., and *Escherichia coli*, along with *Malassezia pachydermatis* and *Candida albicans* (*C. albicans*), naturally inhabit the ear epithelium as commensal microorganisms (Rosales et al. 2024). However, the ability of some of these microorganisms, particularly *Staphylococcus spp*., *Pseudomonas spp*. and *C. albicans*, to form biofilms contributes to increased antimicrobial resistance and impaired immune responses, leading to treatment failures and chronic and recurrent infections (Luciani et al. 2023). The Minimum Inhibitory Concentration (MIC) of currently available antibiotics is measured in the planktonic phase of microorganisms; however, in the biofilm phase, these MIC values can increase up to 1000‐fold (Verderosa et al. 2019).

The treatment strategy for OE typically involves the empirical use of antibacterial, antifungal, and anti‐inflammatory drugs (Chan et al. 2019). However, the suboptimal use of antimicrobial agents in medicine, veterinary care and agriculture has led to an increase in multidrug‐resistant microorganisms (MDROs), including extensively and pan‐drug‐resistant strains (Meshkat et al. 2021, Rafati Zomorodi et al. 2020). In recent years, the assessment of natural products, including essential oils (EOs), has gained significant attention as a novel strategy to address the declining antimicrobial pipeline (Aljaafari et al. 2021). Numerous studies report promising antimicrobial activities of EOs, such as cinnamon against *Porphyromonas gingivalis* (Wang et al. 2018), peppermint EO against the planktonic and biofilm phases of *Staphylococcus aureus* (*S. aureus*) (Kang et al. 2019), lemongrass EO against the planktonic and biofilm phases of *Candida tropicalis* (Sahal et al. 2020), and an oregano EO against *Vibrio vulnificus* (Luo et al. 2022).

Linalool (C_10_H_18_O), a monoterpene alcohol, has been identified as one of the major volatile compounds extracted from over 200 plant species globally (Herman et al. 2016). The antibacterial and antifungal activities of linalool have been established; moreover, this compound also exhibits antioxidant, anti‐inflammatory, and anti‐cancer properties (Gao et al. 2019, Guo et al. 2021, Guo et al. 2021). Additionally, linalool has been recognised as a vital component in domestic products, cosmetic preservatives, food preservatives and the synthesis of vitamins A and E, with an estimated annual usage of 1000 metric tonnes worldwide. However, the application of linalool and essential oils remains challenging due to their high volatility, low utilisation rate and solubility issues (Herman et al. 2016, Zhong et al. 2021).

This investigation aimed to assess the antimicrobial and antibiofilm activity of lavender EO (LEO) against *S. aureus*, *Staphylococcus epidermidis* (*S. epidermidis*), and *C. albicans* isolated from dogs with clinical OE. Additionally, the study compared the antimicrobial and antibiofilm efficacy of LEO with that of linalool.

## 2 Material and Methods

### 2.1 Preparation of LEO and Linalool

The aerial parts of cultivated *Lavandula angustifolia* (batch number 1092) were sourced from Pakan Bazar Company in Isfahan, Iran. Subsequently, the plant was taxonomically identified and catalogued with the herbarium number PMP‐2327 by botanists at the Herbarium of the Faculty of Pharmacy, University of Tehran. To prepare the essential oil, 100 grams of the lavender's aerial parts were washed, dried at room temperature, and then ground into a powder using a grinder. Linalool was purchased from Sigma‐Aldrich (UK; 98%; CAS number STBJ6366). The preparation of LEO was conducted as follows: 50 g of dried lavender plant powder was sampled and suspended in 600 mL of distilled water in a sterile 2 L Erlenmeyer flask. Hydrodistillation was then performed using a Clevenger apparatus for 4 h. Finally, the extracted oil was dehydrated using sodium sulphate and stored in a capped container at 4°C.

### 2.2 Gas Chromatography‐mass Spectrometry (GC‐MS) Analysis

The chemical composition of the LEO was analysed using GC‐MS. An AGILENT 6890 gas chromatograph, coupled with an AGILENT 5973 series mass selective detector, was employed for this purpose. The column used had a length of 30 metres, an inner diameter of 250 mm, and a film thickness of 25 mm. The injector and mass detector temperatures were set at 250°C and 230°C, respectively. The oven temperature programme started at 40°C was held for 1 min, then increased to 250°C at a rate of 3°C per minute, and maintained at 250°C for 20 min. Helium served as the carrier gas at a flow rate of 1.0 mL/min. The oil's compounds were identified by comparing their retention indices (RIs) and mass spectra fragmentation patterns with those in the Wiley 7 n.1 Mass Computer Library.

### 2.3 Microorganisms and Culture Conditions

In this investigation, a total of five clinical microorganisms were included, comprising three clinical isolates of *C. albicans*, two isolates of *S. aureus*, and two isolates of *S. epidermidis*; all isolates were obtained from dogs with otitis. The *C. albicans* isolates were previously identified using the RapID Yeast Plus System (Remel, USA) and were stored as a collection at the Department of Mycology, Veterinary School of Tehran University. Additionally, the *S. aureus* and *S. epidermidis* isolates were obtained from a collection in the Department of Microbiology, Veterinary School of Tehran University. The *C. albicans* ATCC 10331, *S. aureus* ATCC 29213, and *S. epidermidis* ATCC 12228 strains were used as quality controls. All microorganisms were preserved in the Brain Heart Infusion broth (BHI) (Merck, Germany) medium supplemented with 30% glycerol (Merck, Germany) at ‐70°C. The deferrisation of *C. albicans* and *Staphylococcus* isolates was conducted by culturing them on Sabouraud dextrose agar (SDA) (Merck, Germany) supplemented with chloramphenicol (Sigma Aldrich, USA) (50 µg/L) and in Luria–Bertani broth (LB) (Merck, Germany), respectively; also, incubation was performed for 48 h at 30°C and for 24 h at 37°C, respectively.

### 2.4 Biochemical Confirmation of Microorganisms

After the initial incubation, *C. albicans* was confirmed by culturing growth colonies on Candida chrome agar, with plates incubated for 48 h at 35°C. The colonies that appeared green were identified as *C. albicans* for further experiments. Additionally, confirmation of the *Staphylococcus* strains was performed using standard biochemical tests, including Gram staining (showing Gram‐positive cocci), catalase/oxidase tests (both species were +ve /‐ve), coagulase tests (+ve for *S. aureus* and ‐ve for *S. epidermidis*), DNase tests (+ve for *S. aureus* and ‐ve for *S. epidermidis*), and susceptibility to novobiocin (both species were susceptible) (Zomorodi et al. 2024).

### 2.5 Determination of Minimum Inhibitory Concentration of LEO and Linalool Against Planktonic Phase of *C. albicans* and *Staphylococcus* Isolates

The antimicrobial activity of LEO and linalool was assessed against *C. albicans* and *Staphylococcus* isolates in the planktonic phase, as recommended by CLSI guidelines (M60‐Ed1 (CLSI 2020) and M100‐Ed31 (CLSI 2021), respectively). First, the LEO and linalool were suspended in RPMI 1640 medium (Merck, Germany) (pH 7) using 0.1% dimethyl sulfoxide (DMSO) (Merck, Germany) to prepare an initial concentration of 80 mg/mL. The investigated concentration range was adjusted from 0.156 to 80 mg/mL using serial dilution in a 96‐well microplate. The final inoculum size of *C. albicans* was adjusted to 0.5–2.5 × 10^3^ CFU/mL in RPMI 1640 medium. For *Staphylococcus* isolates, the initial inoculum was prepared in trypticase soy broth (TSB; Merck, Germany) at 1 × 10^6^ CFU/mL and then diluted 1:2 in Mueller–Hinton Broth (MHB) to achieve a final concentration of 5 × 10^5^ CFU/mL for MIC testing. The test was conducted using MHB medium (Merck, Germany) for *Staphylococcus* isolates and RPMI 1640 for *C. albicans* isolates. Two wells in each row were designated as positive and negative controls. The positive control was filled with only the medium and the microorganism inoculum, while the negative control contained only the medium and either LEO or linalool suspensions. After overnight incubation at 37°C, the well containing the lowest concentration of LEO or linalool that exhibited no visible growth turbidity was interpreted as the MIC.

### 2.6 Determination of Minimum Bactericidal Concentration (MBC) and Minimum Fungicidal Concentration (MFC)

In continuation of the previous section, 10 µL samples were taken from the MIC wells, as well as from wells containing higher concentrations. These samples were then utilised to perform spread plate cultures on Mueller–Hinton agar (Merck, Germany) for *Staphylococcus* isolates and SDA for *C. albicans*. The plates were incubated at 37°C for 24 h for *Staphylococcus* isolates and at 30°C for 48 h for *C. albicans* isolates to evaluate potential growth. The culture corresponding to the lowest concentration of LEO and linalool, which showed no growth of colonies, was identified as the MBC or MFC.

### 2.7 Assessment of Biofilm Production of *C. albicans* and *Staphylococcus* Isolates

The tissue microtiter plate assay was conducted to demonstrate biofilm production by the tested microorganisms, as previously described (Lohse et al. 2017, Stepanović et al. 2007). Two to three colonies of freshly cultured *C. albicans* and *Staphylococcus* isolates were inoculated into yeast peptone dextrose (YPD) and tryptic soy broth (TSB) (Merck, Germany), both of which were supplemented with 0.5% glucose. After overnight incubation at 37°C, the media were centrifuged at 3000 rpm for 10 minutes; the supernatants were discarded, and the pellets were washed gently twice with phosphate‐buffered saline (PBS, pH 7.2). The pellet cells were resuspended in RPMI 1640 for *C. albicans* and in TSB for *Staphylococcus* isolates, adjusted to 1 × 10^6^ CFU/ml using a hemocytometer slide. Next, 100 µL of each microbial suspension was added to a 96‐well polystyrene microtiter plate, with the exception of column 12, which was designated as the negative control. Following another overnight incubation at 37°C, the medium was aspirated, and the wells were washed three times with PBS. The wells were fixed with 150 µL methanol for 20 min, then stained with 150 µL of 0.1% crystal violet for 10 min before washing. Finally, the dye was resolubilised with 150 µL of 33% glacial acetic acid. The optical density (OD) at 570 nm was measured using an ELISA reader, and biofilm production was reported based on the formula in Table 1.

**TABLE 1:** Interpretation of biofilm potential production.

The formula	Results
OD ≤ OD_C_ ^*^	**No biofilm producer**
OD_C_ < OD ≤ 2 × OD_C_	**Weak biofilm producer**
2 × OD_C_ < OD ≤ 4 × OD_C_	**Moderate biofilm producer**
4 × OD_C_ < OD	**Strong biofilm producer**

Abbreviations: OD_C_, cut‐off OD; OD, optical density; SD, standard division.

* OD_C_ is defined as the average OD of the negative control plus three standard deviations of the negative control.

### 2.8 Anti‐biofilm Activity of LEO and Linalool Against *C. albicans* and *Staphylococcus* Isolates

In this section, the anti‐biofilm effects of different concentrations of LEO and linalool suspensions were separately investigated on *C. albicans* and *Staphylococcus* isolates. As described previously, varying concentrations of LEO and linalool were prepared to a final volume of 100 µL in each well. Subsequently, 100 µL of *C. albicans* and *Staphylococcus* isolate suspensions, each equal to 1 × 10^6^ CFU/mL, were added to the wells, with the positive control containing only the microbial suspension. After incubation at 37°C for 24 h, the percentage of biofilm inhibition was calculated using the following formula: 100–((OD at 560 nm of treated wells) / (mean OD at 560 nm of control wells without antimicrobial agent) × 100) (Esfandiary et al. 2024).

### 2.9 Anti‐biofilm Activity of LEO and Linalool Against Dual Biofilm Producing of *C. albicans* and *Staphylococcus* Isolates

This method represents the anti‐biofilm activity of LEO and linalool separately when exposed to combinations of subjected microorganisms, which were *C. albicans* ATCC 10331 and one clinical *S. aureus* isolates and *C. albicans* ATCC 10331 with clinical *S. epidermidis* isolates. The percentage of biofilm inhibition was calculated as explained above.

### 2.10 Scanning Electronic Microscopic (SEM) Visualisation

The cell and biofilm structures of the tested microorganisms after treatment with LEO and linalool suspension (each applied separately), as well as in the absence of treatment, were demonstrated using SEM. Sample preparation began with placing 7 mm PVC slides into the wells of a 24‐well cell culture plate. Subsequently, 1 mL of LEO and linalool suspension, which was suspended in buffered RPMI‐1640 with 0.1% DMSO at a MIC/2 concentration, was added to each well containing 1 mL of the prepared microorganism suspension as previously described. Additionally, *C. albicans* and *Staphylococcus* isolate suspensions were separately mixed in the following wells with culture medium without LEO and linalool suspension. Furthermore, yeast and bacteria were mixed in a 1:1 ratio and added to wells containing culture medium without LEO and linalool suspension. After an incubation period of 48 h at 37°C, the medium was discarded from the wells, and the slides were gently washed with PBS. Fixation was accomplished by adding 2.5% glutaraldehyde for 2 h at 4°C. The samples were then dehydrated with alcohol in 30%, 70%, 80%, 90% and 95% concentrations. Finally, the samples were coated with a gold layer, and their three‐dimensional structures were captured using a JEOL JSM‐840 scanning electron microscope.

### 2.11 Statistical Analysis

All experiments were performed in triplicate, and the results were expressed as mean ± standard deviations. Statistical analysis was carried out using SPSS version 25.0 (SPSS, Chicago, IL, USA). Data were analysed using one‐way analysis of variance (ANOVA), followed by multiple comparisons between treatments using the Bonferroni test. A *p*‐value of less than 0.05 was considered statistically significant for all analyses.

## 3 Results

### 3.1 GC‐MS Analysis

A total of 37 different components were detected in LEO using GC‐MS analysis. The most predominant component was linalool (42.33%), followed by alpha‐pinene (12.47%), alpha‐terpinolene (8.01%) and limonene (7.89%) (Table 2).

**TABLE 2:** GC‐MS analysis of lavender essential oil.

No	RT (min)	Area%	Name	Quality	CAS Number
1	4.376	0.07	Cyclofenchene	96	000488‐97‐1
2	4.859	0.01	2‐Bornene	96	000464‐17‐5
3	4.963	0.07	Bornylene	95	000464‐17‐5
4	5.131	0.12	(4E)‐2,6‐Dimethyl‐4‐octene	94	062960‐76‐3
5	5.456	0.47	Tricyclene	96	000508‐32‐7
6	6.022	12.47	Alpha‐PINENE	89	000080‐56‐8
7	6.379	4.23	Camphene	98	000079‐92‐5
8	6.955	0.01	Delta‐2‐carene	95	000000‐00‐0
9	7.029	0.01	Linaloyl oxide	62	000000‐00‐0
10	7.207	0.65	Beta‐pinene	97	018172‐67‐3
11	7.464	0.24	Delta‐4(8)‐Menthene	96	001124‐27‐2
12	7.763	0.41	Beta‐Myrcene	96	000123‐35‐3
13	8.009	0.15	p‐Menth‐3‐ene	93	000500‐00‐5
14	8.229	0.44	l‐Phellandrene	97	000099‐83‐2
15	8.449	0.43	Delta‐3‐Carene	97	013466‐78‐9
16	8.869	7.57	Alpha‐Terpinene	98	000099‐86‐5
17	9.241	4.43	Cymene	94	000527‐84‐4
18	9.451	7.89	Dl‐Limonene	97	000138‐86‐3
19	10.053	0.09	Sabinene	93	003387‐41‐5
20	10.509	1.98	Gamma‐Terpinene	97	000099‐85‐4
21	10.955	0.07	(1R)‐(+)‐Trans‐Isolimonene	58	005113‐87‐1
22	11.909	8.02	Alpha‐Terpinolene	98	000586‐62‐9
23	12.963	42.33	Linalool L	97	000078‐70‐6
24	13.062	0.11	Endo‐Isofenchol	91	000534‐35‐0
25	13.157	0.15	D‐Fenchyl alcohol	98	001632‐73‐1
26	14.105	0.39	Dihydrolinalool	80	000000‐00‐0
27	14.383	4.53	Camphor	98	000076‐22‐2
28	14.818	0.93	Isoborneol	96	000124‐76‐5
29	15.159	0.39	BORNEOL L	95	000464‐45‐9
30	15.704	0.02	Beta‐Phellandrene	64	000555‐10‐2
31	16.354	0.61	Alpha‐Terpineol	90	010482‐56‐1
32	17.796	0.01	Carveol	56	000099‐48‐9
33	18.598	0.10	Trifluoroacetyl‐isopulegol	49	028587‐54‐4
34	18.949	0.02	2,5‐Dimethoxytoluene	64	024599‐58‐4
35	20.626	0.35	4,5,6,7‐Tetrahydrophthalide	72	066309‐76‐0
36	23.394	0.03	Linalyl isobutyrate	86	000078‐35‐3
37	24.637	0.01	Geranyl butyrate	80	000106‐29‐6

### 3.2 Determination of MIC and MBC/MFC of LEO and Linalool Against Planktonic Phase of *C. albicans* and *Staphylococcus* Isolates

In general, linalool was more effective against all tested microorganisms in the planktonic phase compared to LEO (Table 3). Interestingly, the MIC of both LEO and linalool for yeast was higher than those for bacteria. Although the MIC of LEO and linalool was similar among *Staphylococcus* isolates, the MIC/MBC ratio for LEO and linalool was 1:2 and 1:1, respectively. In contrast, the MIC/MFC ratio for LEO and linalool was 1:1 and 1:4, respectively.

**TABLE 3:** The MIC and MBC of lavender ES and linalool against C. albicans and Staphylococcus isolates in planktonic phase.

	Lavender ES			Linalool		
Microorganisms	MIC (µg/ml)	MBC/MFC (µg/ml)	Ratio	MIC (µg/ml)	MBC/MFC (µg/ml)	Ratio
*S. aureus* 1	1250	2500	2	1250	1250	1
*S. aureus* ATCC 29213	1250	2500	2	1250	1250	1
*S. epidermidis* 1	1250	2500	2	1250	1250	1
*S. epidermidis* ATCC 12228	1250	2500	2	1250	1250	1
*C. albicans* 1	5000	5000	1	2500	10,000	4
*C. albicans* 2	5000	5000	1	2500	10,000	4
*C. albicans* 3	5000	5000	1	2500	10,000	4
*C. albicans* ATCC 29213	5000	5000	1	2500	10,000	4

Abbreviations: ES, essential oil; MBC, minimum bactericidal concentration; MFC, minimum fungicidal concentration; MIC, minimum inhibitory concentration.

### 3.3 Biofilm Production of Tested Microorganisms

All subjected microorganisms in the present study were biofilm producers. There were only two moderate biofilm producers, including *S. aureus* ATCC 29213 and *C. albicans* 1 (Tables 4 and 5)

**TABLE 4:** The results of biofilm production.

Microorganisms	OD (Mean)	SD	OD of Negative control	Results
*S. aureus* 1	1.82	0.37	0.2	Strong
*S. aureus* ATCC 29213	0.94	0.17	0.25	Moderate
*S. epidermidis* 1	1.44	0.19	0.24	Strong
*S. epidermidis* ATCC 12228	1.13	0.45	0.25	Strong
*C. albicans* 1	0.76	0.07	0.23	Strong
*C. albicans* 2	1.44	0.19	0.27	Moderate
*C. albicans 3*	1.1	0.17	0.28	Strong
*C. albicans* ATCC 29213	0.87	0.10	0.21	Strong

Abbreviations: OD, optical density; SD, standard division.

**TABLE 5:** The MIC and MBC/MFC of lavender ES and linalool against single‐ and dual‐biofilm formation of C. albicans and Staphylococcus isolates.

	Lavender ES		Linalool	
Microorganisms	MIC_90_ (µg/ml)	MBC/MFC_90_ (µg/ml)	MIC_90_ (µg/ml)	MBC/MFC_90_ (µg/ml)
*C. albicans* ATCC 29213 + *S. aureus* 1	2500	10,000	5000	20,000
*C. albicans* + *S. epidermidis* 1	5000	10,000	10,000	20,000
*C. albicans* ATCC 29213	20,000	20,000	40,000	40,000
*S. aureus* 1	5000	20,000	5000	10,000
*S. epidermidis* 1	5000	20,000	5000	10,000

Abbreviations: ES, essential oil; MBC, minimum bactericidal concentration; MFC, minimum fungicidal concentration; MIC, minimum inhibitory concentration.

### 3.4 The Effect of LEO and linalool on Biofilm

Table 4 presents the MIC and MBC/MFC of LEO and linalool against single and dual‐biofilm yeast and bacteria. A comparison of results revealed a consistent ratio of MIC to MBC/MFC (1:4) for both agents against dual‐biofilms of yeast and bacteria, indicating that the MBC/MFC was four‐fold higher than the MIC. Additionally, linalool demonstrated greater effectiveness at higher concentrations compared to LEO.

**FIGURE 1:**
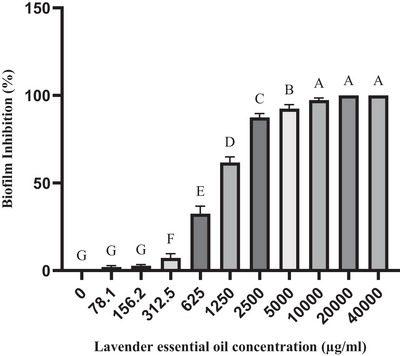
Mean inhibitory effect of lavender essential oil on biofilm formation by *S. aureus* and *S. epidermidis* isolates. Different letters on each column indicate a significant difference from other columns.

**FIGURE 2:**
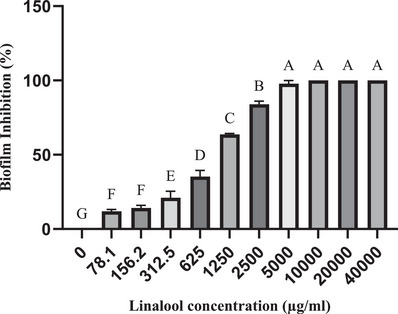
Mean inhibitory effect of linalool on biofilm formation by *S. aureus* and *S. epidermidis* isolates. Different letters on each column indicate a significant difference from other columns.

**FIGURE 3:**
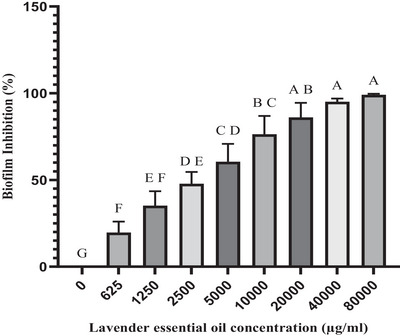
Mean inhibitory effect of lavender essential oil on biofilm formation by *C. albicans* isolates. Different letters on each column indicate a significant difference from other columns.

**FIGURE 4:**
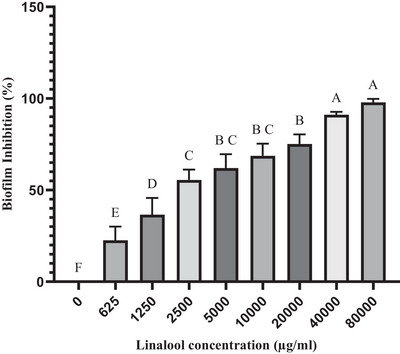
Mean inhibitory effect of linalool on biofilm formation by *C. albicans* isolates. Different letters on each column indicate a significant difference from other columns.

The dose‐dependent effects of LEO and linalool against single biofilms of *S. aureus* and *S. epidermidis* isolates were significant at a concentration of 1250 µg/ml, which inhibited 50% of biofilm formation (*p* ≤ 0.05). Furthermore, a 90% inhibition of biofilm formation was observed at a concentration of 5000 µg/ml. Complete inhibition was achieved at concentrations of 20,000 µg/ml for LEO and 10,000 µg/ml for linalool (Figures 1 and 2). Notably, these values were even higher in the context of biofilm formation by *C. albicans* isolates. Specifically, 50% and 90% inhibition of biofilm formation against *C. albicans* isolates was achieved at concentrations of 10,000 and 40,000 µg/ml for LEO and at 2500 and 40,000 µg/ml for linalool, respectively (Figures 3 and 4)

**FIGURE 5:**
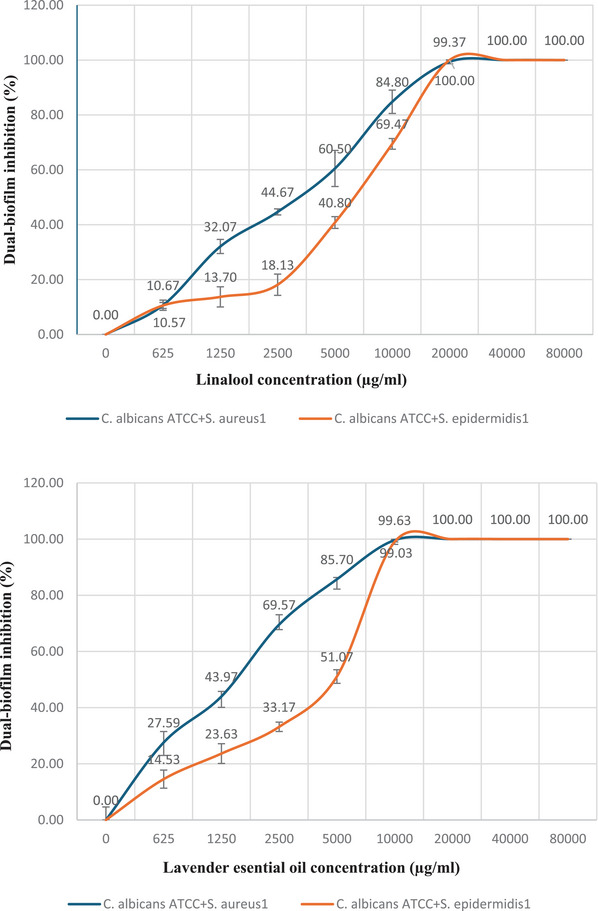
Inhibitory effect of lavender essential oil and linalool on dual biofilms formed by *C. albicans* + *S. aureus* and *C. albicans* + *S. epidermidis*.

The LEO demonstrated a 50% inhibition of dual biofilms formed by *C. albicans* + *S. aureus* and *C. albicans* + *S. epidermidis* at lower concentrations compared to linalool. As illustrated in Figure 5, LEO at concentrations of 2500 and 5000 µg/ml inhibited the biofilm of *C. albicans* + *S. aureus* by 69.57% ± 1.8 and that of *C. albicans* + *S. epidermidis* by 51.07% ± 2.4, respectively. In contrast, linalool exhibited inhibition of the dual biofilm of *C. albicans* + *S. aureus* at a concentration of 5000 µg/ml, achieving an inhibition rate of 60.5% ± 1.8, and inhibited the *C. albicans* + *S. epidermidis* biofilm at a concentration of 10,000 µg/ml with an inhibition rate of 69.47% ± 1.8.

### 3.5 SEM Analysis

**FIGURE 6:**
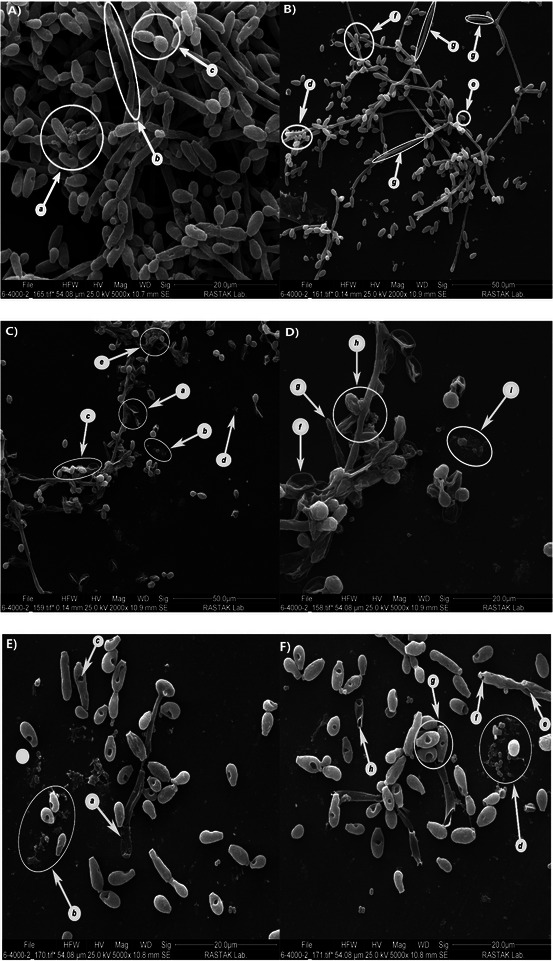
SEM images showing various dual biofilms of *C. albicans*/*S. aureus* **(A)** and *C. albicans*/*S. epidermidis* **(B)** as control groups. **(C, D)** depict the dual biofilms of *C. albicans*/*S. epidermidis* and *C. albicans*/*S. aureus*, respectively, after treatment with lavender essential oil at MIC/2 concentration. Disruption of integrity and pore formation are evident among the hyphae (arrows e, g); ballistoconidia were observed with reduced cell accumulation, increased cell wrinkling, and membrane disruption (arrows a, c, h, f). Disturbances in bacterial cells are indicated (arrows b, d, i). **(E, F)** show dual biofilms of *C. albicans*/*S. aureus* and *C. albicans*/*S. epidermidis*, respectively, following treatment with linalool at MIC/2 concentration. Notable disruptions and cell membrane lysis of hyphae are observed (arrows a, h, e, f), along with pore formation on the cell membrane of *C. albicans* (arrows c, b, g). Bacterial cells appear disturbed and detached from the yeast structures (arrows b, d).

As demonstrated in captured photos from control samples (Figure 6), a high density of dual biofilm consisted of *C. albicans* hyphae (Figure 6: A and B; arrows g, b) and ballistoconidia (Figure 6: A and B; arrows c, e) that *Staphylococcus* isolates were attached to the hyphae (Figure 6: A and B; arrow d) or ballistoconidia (Figure 6: A and B; arrows a, f). Treatment with MIC/2 concentration of LEO generally reduced the density of biofilms, certainly hyphae structures. Also, the morphology of *Staphylococcus* isolates was deformed and cell accumulation was decreased. In comparison, treatment with linalool in MIC/2 concentration revealed higher anti‐biofilm activity against yeast and bacteria.

## 4 Discussion

OE is the most frequently diagnosed condition in pet animals, particularly in dogs. Factors contributing to the persistence of otitis once established, which necessitates treatment to prevent recurrence, include those associated with chronic diseases, such as chronic inflammation and the progressive pathological changes within the ear canal and associated otitis media (Tambella et al. 2020). However, biofilm formation by certain microorganisms, such as *S. aureus*, *S. epidermidis* and *C. albicans*, can lead to tolerance to antimicrobial agents and subsequent treatment failure (Nocera et al. 2023). Therefore, a new approach that includes the use of EOs with appropriate antimicrobial and antibiofilm activity has recently been highlighted. In the present study, the antimicrobial and antibiofilm activity of the LEO was assessed against *S. aureus*, *S. epidermidis* and *C. albicans* isolates.

The LEO consists of linalool in a range of 20% to 45%, as reported by ISO (Lis‐Balchin and Hart 1999). This finding supports our GC‐MS analysis of the LEO, which identified linalool as the most prevalent component, with a frequency of 42.33% among 37 different identified chemical compounds. Additionally, Valkova et al. found linalool to be the most common component in their study of 44 identified chemical compounds in LEO, with a frequency of 32.7% (Valková et al. 2021). However, these findings contradict those of Puvaca et al., who reported a linalool frequency of only 10.71% in LEO (Puvača et al. 2021). Another frequently identified component in the present study was α‐pinene (12.47%), along with α‐terpinolene (8.01%), limonene (7.89%), and camphor (4.53%), all of which were higher than the values published by ISO (α‐pinene, 0%; α‐terpinolene, 0–2%; limonene, 0–1%; camphor, 0–1.5%). However, the amounts of β‐phellandrene (0.02%) and α‐terpineol (0.61%) were in agreement with the ISO values (0‐1% and 0–2%, respectively) (Lis‐Balchin and Hart 1999).

In the present survey, LEO demonstrated a growth inhibitory effect against *S. aureus* and *S. epidermidis* at an MIC of 1250 µg/ml, while the MIC for *C. albicans* isolates was 5000 µg/ml. The MBC and MFC of LEO were determined to be 2500 µg/ml and 5000 µg/ml, respectively. Furthermore, the antimicrobial activity of linalool, the most prevalent compound in LEO, was evaluated. The MIC and MBC values for linalool against *Staphylococcus* isolates were found to be 1250 µg/ml and 2500 µg/ml, respectively. In contrast, the results for *C. albicans* differed, with MIC and MFC values of 2500 µg/ml and 10,000 µg/ml, respectively. According to the systematic literature review, the antimicrobial activity of EOs has been well established, with LEO being extensively investigated (Aprotosoaie et al. 2017, Malloggi et al. 2022). Cruz Sanchez et al. determined that increasing the concentration of LEO significantly enhanced its inhibitory effect against *S. aureus*, *S. epidermidis* and *C. albicans* strains. Additionally, they tested LEO at various concentrations on biosynthetic membranes and did not find significant differences (Cruz Sánchez et al. 2024). However, several studies have indicated that using LEO coated on different biosynthetic membranes increases its inhibitory effect due to the antimicrobial activity of the tested membranes (Danila et al. 2021, Liu et al. 2022, Sukhodub et al. 2023).

The lower determination of the MIC and MBC/MFC of LEO in some experiments compared to linalool may be attributed to the presence of other compounds. For instance, the antimicrobial activity of α‐pinene, which is the second most prevalent compound in the current study, has been observed in previous investigations (Leite‐Sampaio et al. 2022, Nóbrega et al. 2021). However, linalool is a major compound found in lavender, basil, and other essential oils known for their antimicrobial activity (Mahizan et al. 2019).

The mechanism of antimicrobial activity of linalool was previously demonstrated by Yang et al., who stated that linalool disrupts cell membrane permeability by inducing oxidative stress. This finding was recorded by measuring the zeta potential of the tested *K. pneumoniae* isolates; treatment with linalool increased the zeta potential to ‐7.83 compared to the non‐treated condition, which had a value of ‐12.1 (Yang et al. 2021). SEM images captured in the current study revealed significant disruption of the cell membrane in the tested isolates after exposure to linalool and LEO.

Interestingly, we observed the antimicrobial activity of linalool and LEO in both the single‐ and dual‐biofilm phases of *Staphylococcus* and *C. albicans* isolates. Generally, the MIC and MBC/MFC ratio of LEO and linalool against *Staphylococcus* isolates in the biofilm phase was 1:2 and 1:1, respectively, while these values against *C. albicans* isolates were 1:1 and 1:4, respectively. Our observations indicated that linalool exhibited higher antimicrobial activity compared to LEO against *Staphylococcus* isolates, whereas LEO was more effective than linalool against *C. albicans* isolates in the biofilm phase. These findings do not confirm the earlier investigation by Brożyna et al., which indicated that LEO emulsions is inactive against *S. aureus* isolates in the biofilm phase (Brożyna et al. 2021). However, a recently conducted systematic review indicated that emulsified LEO with Tween 20 did not exhibit antibiofilm activity, while non‐emulsified LEO was able to eradicate biofilms of *S. aureus* in the range of 40% to 70% (Truong and Mudgil 2023). This discrepancy may be explained by differences in sample size, the characteristics of the tested microorganisms, the methodology for extracting EOs, and variety of used lavender types.

Although LEO was effective against all microorganisms tested in the current study, *C. albicans* isolates exhibited greater resistance to LEO. In general, there are few mechanisms identified as exhibiting antibiofilm activity of EOs in fungi, which include fungicidal effects, prevention of adhesion, disruption of intracellular connections, and modulation of morphogenesis in dimorphic fungi (Budzyńska et al. 2017). The biofilm inhibition of LEO among *C. albicans* isolates at the MIC was estimated to be approximately 60%. This value is lower than the previous findings, which reported a 75% –80% inhibition of biofilm growth of *C. albicans* at the MIC of LEO (Karpiński et al. 2023, Santos et al. 2024).

Investigating the antibiofilm activity of LEO in dual biofilms formed by fungi and bacteria is one of the few studies conducted in this area. Our results demonstrated that LEO completely inhibited the dual biofilms formed by *C. albicans*/*S. aureus* and *C. albicans*/*S. epidermidis* at a concentration of 20,000 µg/mL. Furthermore, the synergistic effect of essential oils in the dual biofilm phase of *C. albicans*/*S. aureus* against various antibiotics and antifungal agents has been evaluated. Li et al. demonstrated the synergistic effect of clove oil when combined with fluconazole or mupirocin against the dual biofilm of *C. albicans*/*S. aureus* (Li et al. 2015).

Limitations highlight the challenges associated with collecting data on the expression of biofilm‐mediated genes following exposure to LEO. Also, there is a need for a comprehensive evaluation of the antimicrobial and antibiofilm activity of LEO against Gram‐negative bacteria. Furthermore, further investigations are warranted to assess the antimicrobial and antibiofilm efficacy of essential oils, particularly LEO, in combination with various antibiotics and antifungal agents. Additionally, the evaluation of certain antibacterial and antifungal agents as controls could be used to compare the antimicrobial and antibiofilm activities of LEO and other essential oils.

## 5 Conclusion

In summary, LEO has exhibited antimicrobial and antibiofilm activity against *S. aureus*, *S. epidermidis*, and *C. albicans* isolates in both planktonic and biofilm phases. Our findings are promising, suggesting the potential of LEO in novel therapeutic approaches to combat the threat of antimicrobial resistance. However, further in vivo investigations are necessary to evaluate the effective dosage of LEO and its potential toxic effects before it can be considered a viable therapeutic option for humans and/or animals.

## Author Contributions

**N.N**.: conceptualization, data curation, funding acquisition, investigation and methodology. **A.A**. and **B.N.F**.: conceptualisation, data validation, formal analysis, project administration and supervision. **S.A**. and **A.A**.: investigation, review and editing of the manuscript and visualisation. **A.R.Z**.: conceptualisation, writing the draft of the manuscript and formal analysis. **J.M**.: conceptualization, data curation and visualisation.

## Conflicts of Interest

The authors declare no conflicts of interest.

## Ethics Statement

The experiment was conducted on archived isolates; therefore, no ethical approval was required.

### Peer Review

The peer review history for this article is available at https://www.webofscience.com/api/gateway/wos/peer‐review/10.1002/vms3.70407.

## Supporting information



Supporting Information

## Data Availability

The datasets generated and analysed during this research are available by contacting the corresponding author.
