# Correction to “Evaluating the Antimicrobial and Antibiofilm Efficacy of Lavender Essential Oil and Linalool on Dual *Candida albicans* Biofilms With S*taphylococcus aureus* and *Staphylococcus epidermidi*s from Canine External Otitis”

**DOI:** 10.1002/vms3.70457

**Published:** 2025-06-26

N. Neisari, A. Sharifzadeh, B. N. Fasaei, et al., “Evaluating the Antimicrobial and Antibiofilm Efficacy of Lavender Essential Oil and Linalool on Dual *Candida albicans* Biofilms With *Staphylococcus aureus* and *Staphylococcus epidermidis* From Canine External Otitis,” Veterinary Medicine and Science 11 (2025): e70407, https://doi.org/10.1002/vms3.70407.

We have identified typographical errors in the article that we would like to correct:

1. On page 2 of 12, in Section 2.3, the strain “*C. albicans* ATCC 10331” was mistakenly cited. The correct strain should be “*C. albicans* ATCC 10231.”

2. In Tables 3, 4, and 5, the ATCC number for *C. albicans* is also incorrectly listed as ATCC 25913. These should all be corrected to ATCC 10231.

We apologize for these errors.

