# Supplementary material for: Evaluating the Antimicrobial and Antibiofilm Efficacy of Lavender Essential Oil and Linalool on Dual Candida albicans Biofilms With Staphylococcus aureus and Staphylococcus epidermidis From Canine External Otitis
Source: Vet Med Sci. 2025 May 23;11(3):e70407. doi: 10.1002/vms3.70407 (PMC12099306; doi:10.1002/vms3.70407)
Supplement: Supplementary file 1 — Supporting Information [file VMS3-11-e70407-s001.docx]

**Author's additional information:**

1. Navid Neisari, Address: University of Tehran, Azadi St., Tehran, Iran, P.O. Box: 14155-6619, Tell: +982161117151, Email: navidneisari@gmail.com, ORCID's ID: https://orcid.org/0000-0002-3702-3586

2. Aghil Sharifzadeh, Address: University of Tehran, Azadi St., Tehran, Iran, P.O. Box: 71348-14336, Tell: +982161117151, Email: asharifzadeh@ut.ac.ir, ORCID's ID: https://orcid.org/0000-0001-6442-0053

3. Bahar Nayeri fasaei, Address: University of Tehran, Azadi St., Tehran, Iran, P.O. Box: 71348-14336, Tell: +982161117151, Email: nayerib@ut.ac.ir, ORCID's ID: https://orcid.org/0000-0003-2373-4667

4. Sepideh Asadi, Address: Amol University of Special Modern Technologies, Abazar 35 Alley, Taleghani Blvd, Amol, Mazandaran, Iran, P.O. Box:46156-64616, Tell: +981144442137, Email:

sepidehasadi1394@yahoo.com, ORCID's ID: https://orcid.org/0000-0002-3659-6745

5. Alireza khosravi, Address: University of Tehran, Azadi St., Tehran, Iran, P.O. Box: 71348-14336, Tell: +982161117151, Email: khosravi@ut.ac.ir, ORCID's ID: https://orcid.org/0000-0002-6777-5930

6. Abolfazl Rafati Zomorodi, Address: Shiraz University of Medical Sciences Z and St., Shiraz, Iran, P.O. Box: 71348-14336, Tell: +987132305410, Email: rafatiabolfazl@gmail.com, ORCID's ID: https://orcid.org/0000-0002-8457-4057

7. Javad Malakootikhah, Address: University of Tehran, Azadi St., Tehran, Iran, P.O. Box: 71348-14336, Tell: +982161117151, Email: j.malakootikhah@nanoageco.ir, ORCID's ID: https://orcid.org/0000-0002-3262-012X
